# Measurement of Prosocial Tendencies: Meta-Analysis of the Generalization of the Reliability of the Instrument

**DOI:** 10.3390/healthcare11040560

**Published:** 2023-02-13

**Authors:** Natalia Reig-Aleixandre, Javier Esparza-Reig, Manuel Martí-Vilar, César Merino-Soto, José Livia

**Affiliations:** 1 Departamento de Humanidades, Universidad Francisco de Vitoria, 28223 Madrid, Spain; 2 Departamento de Psicología, Universidad Europea de Valencia, 46010 Valencia, Spain; 3 Departamento de Psicología Básica, Universitat de València, Avgda. Blasco Ibañez 21, 46010 Valencia, Spain; 4 Instituto de Investigación de Psicología, Universidad San Martín de Porres, Lima 34, Peru; 5 Facultad de Psicología, Universidad Nacional Federico Villarreal, Lima 15088, Peru

**Keywords:** generalization of reliability, measurement of prosocial tendencies, measurement of prosocial behavior, reliability, Cronbach’s alpha, systematic review

## Abstract

The Prosocial Tendencies Measure (PTM) and its revised version (PTM-R) are used internationally to measure prosocial behaviors in different life situations. To obtain accumulated evidence of the report and the reliability of its scores, a meta-analysis of the reliability of internal consistency was performed. The databases of Web of Science (WoS) and Scopus were reviewed and all the studies that applied it from 2002 to 2021 were selected. Results: Only 47.9% of the studies presented the index of reliability of PTM and PTM-R. The meta-analytic results of the reliability report of the subscales that the PTM and the PTM-R have in common were: Public 0.78 (95% CI: 0.76–0.80), Anonymous 0.80 (95% CI: 0.79–0.82), Dire 0.74 (95% CI: 0.71–0.76), and Compliant 0.71 (95% CI: 0.72–0.78). Each one of them presents high levels of heterogeneity derived from the gender of the participants (percentage of women), the continent of the population, the validation design, the incentive to participate, and the form of application. It is concluded that both versions present acceptable reliabilities to measure prosocial behavior in different groups and situations, as adolescents and young people, but their clinical use is discouraged.

## 1. Introduction

In general terms, prosocial behaviors refer to all kinds of actions that benefit others and that are carried out voluntarily. People who engage in prosocial behavior enjoy helping others [1]. These behaviors promote the productivity of organizations, improve the wealth of societies and, above all, improve the health and quality of life of the people who perform them. Corresponding to this importance and pervasiveness, human prosociality has received considerable attention in some scientific disciplines, including medicine, biology, sociology, and, obviously, psychology [2].

### 1.1. Prosocial Behaviors and Health

The relationship between prosocial behaviors and health has been extensively studied, particularly in non-clinical samples, due to the ease of finding samples with these characteristics. For example, Schacter and Margolin [3] suggested that daily helping behaviors can meet the social and emotional needs of depressed youth and are negatively correlated with a depressed mood [4]. In turn, this type of prosocial behavior was a predictor of a decrease in gambling addiction [5]. On the other hand, Miles et al. [6] recommended promoting prosocial behavior in times of catastrophe to safeguard mental health and foster a positive emotional state. Regarding the relationship between prosocial behaviors and the health status of youth, a study with Spanish university students concludes that prosociality has positive effects on the perception of satisfaction with life [7]. Likewise, some results conclude that promoting prosocial behaviors among adolescents who have experienced traumatic situations is associated with greater resilience [8].

The benefits of prosocial behaviors not only concern psychological health but also health in general. Some studies show the relationship between these behaviors and neurohormonal circuits (especially oxytocin and progesterone) that have a buffering effect on stress and restorative properties of the organism [9]. There is also evidence that people who informally help others experience positive mental states associated with psychological well-being, good health, and longevity [10,11,12]. In professional practice, prosocial behaviors toward patients allow nurses to feel less fatigue and work with greater vitality, despite the heavy workload [13]. On the other hand, adolescents with low prosocial behavior associated with relationship callous traits lead to greater self-reported mental health difficulties in young adulthood [14]. These empirical findings strengthen the theory of the influence of prosocial behavior on variables that improve the person’s adaptability to their social and professional environment.

### 1.2. Measurement of Prosocial Behaviors

Prosocial behavior includes a wide range of specific behaviors, which encourages debates about how to measure it and determine what its components are [15]. This wide range of behaviors can be deduced from multidimensional instruments that measure prosocial behavior, in which moderate or low correlations suggest that the constructs measured are associated, but completely independent.

The measurement of prosocial behavior implies having instruments that gather solid evidence of validity and reliability in a wide range of aspects. In a recent systematic review [16], 16 instruments that were considered relevant to this construct were identified. It allowed an organized and updated knowledge basis. This identification of essential aspects will make it possible to carry out other studies and provide information so that researchers can apply relevant instruments. However, the structuring of the validity evidence using a consensus framework of validity theory was absent. Following a good framework would have facilitated identifying the sources of validity of these instruments, and thus ensuring the interpretation and use of the scores of measures of prosocial behavior [17]. In particular, and linked to the objective of this study, the precision of the scores also requires attention, since this property indicates the degree of measurement error contained in the scores [17].

Given the importance of understanding and, above all, measuring the various behaviors and circumstances that come with prosociality, Carlo and Randall [18] built the Prosocial Trends Measure (PTM). A 23-item scale composed of six subscales: public prosocial behavior (4 items), emotional prosocial behavior (4 items), emergency prosocial behavior (3 items), altruistic prosocial behavior (5 items), anonymous prosocial behavior (5 items), and prosocial behavior of compliance or obedience (2 items). Carlo and Randall [18] created this multidimensional scale because they were not convinced of the idea that prosocial behaviors were a global behavior category [19]. The response scale was established in a range from 1 (does not describe me at all) to 5 (describes me a lot). Likewise, its internal structure was evaluated with an exploratory factor analysis that identified six factors that explained 63.38% of the variance. The item–test correlations in each dimension showed values above 0.40. Regarding the evidence of discriminative validity between the dimensions of the PTM, the correlations ranged between low magnitudes and some averages. The Public subscale showed negative correlations with Anonymous, Emotional, Pleasure, and Altruism. It also had direct coefficients among all the other subscales, with no significant relationship between public and anonymous, anonymous and altruism, and emergency and altruism.

The internal consistency of the scale scores, measured by the *α* coefficient ranged from 0.74 to 0.85, with gender differences in some dimensions. On the other hand, the criterion validity was evaluated, with positive and significant correlations above 0.20. A complementary study by [18] made it possible to examine the stability of the test–retest scores, in addition to evaluating the relationships of the PTM with other measures of prosocial behaviors.

The results of [18,20,21,22] showed that although the subscales were expected to correlate positively and moderately, in most cases the correlations were weak or non-significant and sometimes even negative. This leads to the idea that prosociality is not a single behavior, but a group of different behaviors [19].

The PTM was modified to be used with early and middle adolescents. For that purpose, a focus group consisting of 10 adolescents between 11 and 16 years old was formed to evaluate the items of the original PTM in terms of clarity and relevance [20]. This allowed the items to be written in simpler language, incorporating two new reagents (one for the prosocial emotional behavior subscale and the other for the prosocial altruistic behavior subscale), constituting a new version of 25 questions (PTM-R). The internal consistency of each subscale of the instrument was examined, the coefficients for average adolescents ranging between 0.75 and 0.86; and from 0.59 to 0.86 for early adolescents. The test–retest reliability range, after 2 weeks, was from 0.56 to 0.82 for middle adolescents and from 0.54 to 0.76 for early adolescents. In general, the PTM-R scales were significantly related to variables such as sympathy, perspective taking, moral reasoning; and not significantly with theoretically irrelevant variables such as vocabulary skills, social desirability, personal anguish.

Carlo et al. [20] evaluated the effects of age and gender on prosocial tendencies. Regarding altruistic prosocial tendencies, both variables were predictive, indicating that middle adolescents and females were more likely to report this aspect than early adolescents and males. Boys were likelier to report these tendencies for public prosocial tendencies, and prosocial emotional tendencies in the case of girls. Regarding anonymous prosocial tendencies, middle adolescents were more likely to report them than early adolescents.

The validation studies of the PTM present some inconsistencies in the report. Thus, the PTM has been translated into six languages: Turkish [23], Chinese [24], Spanish [25], Persian [26], Greek [27], and Serbian [28]; and the PTM- R has been translated into two languages: Portuguese [29] and German [19]. The PTM was used by [30] with an Italian sample; however, they did not provide data on how the translation into Italian was made. The Turkish and Chinese studies only present the abstract in English, the rest of the article is written in their respective languages. In most of the studies, they added new items to the original version, and it was only in the Spanish, Greek, and Serbian translations that the number of items was preserved. In these studies, the validity of the structure converges in the multidimensional model originally designed by its authors (i.e., six dimensions), as it was indicated in their confirmatory factor analysis (CFA). Only four of these studies report reliability, since the rest do not report it or induce it from other studies. In these validation studies, the size of the alpha coefficients for the subscales is established in a range from 0.86 to 0.54.

In both instruments, as it is the case of others that also evaluate prosocial behavior, the research that synthesize their characteristics and describe their correlates have not managed to systematically review their specific psychometric properties, and meta-analyze some of these parameters (e.g., reliability, factor loadings, etc.). The accumulated and organized evidence on the psychometric properties of an instrument helps to make decisions about the use and interpretation of its scores, in the context of its limitations. Therefore, a systematic review of these psychometric parameters can provide significant answers to the quality of a measure. The systematic review (SR) seeks to collect, critically evaluate, and synthesize the results of multiple primary studies, which enables the creation of a body of knowledge on a given topic [31]. On the other hand, meta-analysis research consists of integrating the numerical results of a set of studies on the same topic, following the rules of scientific rigor to obtain a global clarification of that topic [32]. When this meta-analytic research is focused on the reliability of the scores of a measure instrument, then the underlying focus is the quality of the instrument. This quality, derived from the precision of its scores, conditions the use of the instrument, as well as the quality of the evidence of the measurement validity and the substantive research with this instrument.

In particular, reliability is a metric attribute of the scores that can be conceptualized as reproducibility, consistency, or precision. It imposes a limit on the validity of the measures, as well as on the use of the scores, due to the amount of error of measurement around the scores. For example, linear correlations between constructs are attenuated to a degree that depends on the amount that the internal consistency reliability deviates from 1.0 [33,34]. Furthermore, the accuracy of the scores to describe a behavior, operationalized by a confidence interval of variation by error, covary directly with the reliability [33,34]. Finally, the differences between scores with clinical value are larger as the error of measurement increases [35], which makes it difficult to interpret the scores to make decisions. 

A change in the conceptualization of reliability has established that reliability is not a property of the scale [36], but rather a characteristic that depends on each application. The reliability of the scores of a scale varies in successive applications. This variation will increase as the differences between the populations from which the sample is drawn are accentuated. There is an incorrect research practice that consists of referring to a reliability that comes from some previous application of the test and not from the current sample. Vacha-Haase et al. [37] named it “reliability induction”, which occurs when reliability coefficients from previous studies are cited. This induces the user to mistakenly believe that the data in question are reliable. However, it is assumed that reliability induction is better than not reporting reliability [38]. In another study, Vacha-Haase et al. [39] already pointed out that a third of the articles reviewed did not mention reliability, that only 36% of the articles provided reliability coefficients for the data analyzed, and 23% induced reliability by suggesting a modification of journal editorial policies to effect change in scoring consistency reporting practices.

### 1.3. The Present Study

The following objectives are pursued in this research:

(a) To analyze the characteristics of the reliability report of the PTM and PTM-R scores. The aim is to estimate the percentage of studies that do not report reliability, the percentage that do not report a value but a reliability range, and the percentage of studies that report induced reliability, that is, from a previous study. As a whole, this characterization is linked to the quality of the studies that report the internal consistency of the PTM.
(b) To estimate meta-analytically the reliability of the Prosocial Tendencies Measure (PTM) and Prosocial Tendencies Measure-Revised (PTM-R) subscales, given the strong multidimensional nature of the instrument. There are 6 subscales, but only the public, anonymous, dire, and compliant subscales will be analyzed, since the other two subscales do not contain the same number of items in both versions of the instrument. It also seeks to examine the sources of variability in the samples that affect the reliability indices of the PTM and the PTM-R.

A meta-analysis of the generalization of reliability is carried out to achieve the objectives. The whole process was performed following the recommendations of the PRISMA guidelines for systematic reviews [40], and the good practice recommendations of reliability generalization meta-analysis studies (REGEMA [41]).

## 2. Materials and Methods

### 2.1. Search and Identification of Studies

**Figure 1:**
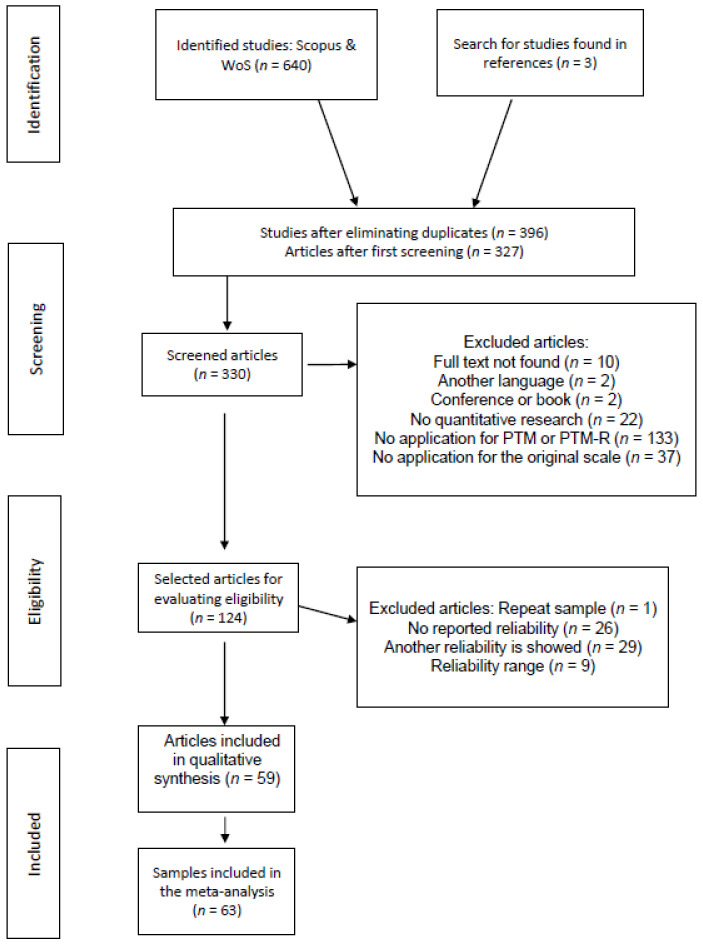
Flow chart of articles included in the meta-analysis.

Firstly, a search was carried out in both the Trip and Cochrane databases, to check if previous systematic reviews or meta-analyses of this topic had been done. This review was performed on 15 June 2021, and no similar studies on the topic were found. Secondly, a search on the Web of Science (WoS) and Scopus databases was carried out, dated 18 June 2021. These were the first databases chosen to start the research, as they are the most used. “Prosocial tendencies measure” or “PTM” was entered as a keyword in the search engine, specifically in the topic section (title, keywords and abstract). A total of 640 sources were identified, 319 in WoS and 321 in Scopus. The studies matched in the two databases and therefore no further search was carried out in other databases. All these studies were entered into the Refworks bibliographic manager. The steps carried out are reflected in Figure 1.

### 2.2. Screening

In the first place, 244 duplicates were eliminated, the remaining articles (*n* = 396) were screened and only articles (not conferences or book chapters) written in English and Spanish were chosen. In total, 327 articles were obtained. From the references of these articles, 3 more articles were subsequently included as it was concluded they were potentially valid. Despite drawing a filter, the bibliographic manager could not correctly detect all the studies and included some written in another language and some that were not articles. These studies were eliminated in subsequent phases.

Second, the abstracts of the studies found were read and a series of inclusion and exclusion criteria were established according to the objectives of the research. The inclusion criteria were as follows: (a) Experimental or quasi-experimental studies. (b) Research published in Spanish or English. (c) Research that applied the PTM or/and the complete PTM-R in its original version, including any validation or translation that did not alter the number of items or their content. (d) Investigations that have correctly indicated the reliability index (Cronbach’s α and/or McDonald’s *ω*) extracted from the study sample itself, that is, investigations that after applying the PTM carried out reliability analyses on their sample and reported the results correctly. (e) Research that communicated the sample size (*n*).

The exclusion criteria during the selection of the study were: (a) non-quantitative or literature review studies, (b) research in other languages, since the main research is written in English, (c) research that did not apply the PTM or PTM-R, (d) investigations that applied a version of the PTM or PTM-R different from the original, and (e) investigations that reported another reliability estimator different from that of the analyzed sample (induced reliability) or give a range (it is imprecise).

After the screening process, 124 articles were obtained.

### 2.3. Eligibility

Third, the full text of each article was read to fully apply the inclusion and exclusion criteria. After the eligibility process, 59 articles were obtained for our study, 41 of which applied the PTM and 18 the PTM-R.

### 2.4. Inclusion

Since three articles presented more than one sample, there were 63 different samples: 44 in which the PTM was applied and 19 in which the PTM-R was applied (Appendix B).

A random effects statistical model was used to calculate the mean value of α using the restricted maximum likelihood estimation (REML) method. Cronbach’s α was the statistic used to measure reliability since McDonald’s *ω* was only used in one article. Of the 63 samples, in 13 of them, only Cronbach’s α was calculated for the full scale, in 44 of them Cronbach’s α was calculated for the subscales, and in 6 of them, Cronbach’s α was calculated for both the sub-scales as well as the global scale. The altruistic and emotional subscales have different numbers of items in the PTM and the PTM-R, so they will be left out of our study. The main statistics of the studies included in the meta-analysis are shown in Table 1.

### 2.5. Coding

The meta-analytic study of the reliability of the PTM and PTM-R, in addition to seeking the estimation of the global reliability of the scale, aims to analyze its variability. In this sense, it is important to choose those moderating variables that can explain, to some extent, the variability in the reliability coefficients. Three groups of variables are considered to explain this variability in the coefficients [32]: methodological factors (such as versions and adaptations of the test, ways of applying it, group size); factors related to the origin and composition of the group (for example, distribution by gender, socioeconomic status, educational level); contextual factors (for example, the objective of the study, year in which it was carried out or published, country or continent in which it was carried out). An Excel record was created and the characteristics of the 63 included studies that may explain part of the variability in the reliability coefficients were collected in it. The variables that were coded are: year of publication of the article, version (PTM or PTM-R) and language of the scale, country and continent in which the PTM was applied, format (applied or self-applied), and form (paper or online) to apply it, mean and standard deviation of the age, mean and standard deviation of the PTM scores, gender of the sample participants (percentage of men and percentage of women).

## 3. Results

### 3.1. Reliability Report

The articles selected in the systematic review were analyzed based on the first objective of this study, which corresponds to the information offered by the authors regarding the recording of reliability scores. Of the studies, 79.04% reported the reliability index: of them, reliability was reported through a range in 7.56% of the cases, reliability was induced from previous research in 23.58% of the studies, and only 47.9% presented adequate reliability. 20.96% of the studies did not report it.

### 3.2. Generalization of Reliability

A reliability generalization meta-analysis was performed over a total of 41 articles for the Public scale, 39 for Anonymous, 38 for Dire, and 41 for Compliant. These studies applied the PTM or the PTM-R and presented the α values for the total scale and the subscales: public, anonymous, dire, and compliant.

The reliability generalization meta-analysis and the calculation of heterogeneity for the scores of the four abovementioned subscales was conducted. The results of these operations are shown in Table 2.

Publication bias was analyzed by performing an Egger test. The results of this test verified that there were no biases in terms of selection, *t*(17) = −1.4431, *p* = 0.1672.

The four presented values of statistically significant heterogeneity, measured with the *Q* value, and a high proportion of variability, measured with the *I^2^* index, were observed. The values for the public subscale were obtained based on the 41 articles in which it appeared, indicating a mean of *α* of 0.78 (95% CI: 0.76–0.8); *Q* (df = 40) 341.56, *p* < 0.01; *I^2^* = 89.51. The mean of α for the anonymous subscale for the 39 studies in which it was included was 0.8 (95% CI: 0.79–0.82), *Q* (df = 38) 240.11, *p* < 0.01; *I^2^* = 87.11. Regarding the dire subscale, the mean of *α* for the 38 items was 0.74 (0.71–0.76), *Q* (df = 37) 366.79, *p* < 0.01; *I^2^* = 89.33. Finally, by calculating the values of the 41 studies in which the compliant subscale appeared, an *α* mean of 0.75 (0.72–0.78) was obtained, *Q* (df = 40) 395.6, *p* < 0.01; *I^2^* = 90.55.

### 3.3. Moderator Analysis

Once the high levels of heterogeneity were observed, an analysis of the variables that could be acting as moderators was carried out. These variables were taken as independent variables, being the value of α the dependent variable. First, a linear meta-regression analysis was performed to calculate the influence of the continuous moderating variables on the α mean. This operation was performed for each of the subscales. The results of these operations appear in Table 3.

The values of age relative to the mean and the standard deviation acted as predictors of the α values according to the analysis of the moderators of the anonymous subscale. The mean age explained 27.1% (*p* < 0.001) of the variance of the heterogeneity and the standard deviation, 13.79% (*p* < 0.05). In both cases, the higher the age score, both in the mean and the standard deviation, the greater the heterogeneity. For the dire subscale, the percentage of women was a predictor of the α value, explaining the variance of heterogeneity at 12.87% (*p* < 0.05). The higher the percentage of women, the greater the heterogeneity. Finally, in the compliant subscale, it was observed that the mean age and the mean score acted as moderators of the *α* values, explaining 26.99% (*p* < 0.0001) and 26.64% (*p* < 0.05) of the variance of heterogeneity, respectively. Again, the higher score on both variables indicated greater heterogeneity. No values that would indicate that the variables acted as moderators of the α values were observed in the public subscale.

Second, the possible influence of the categorical variables on the α values was analyzed using an analysis of variance (ANOVA). In this case, it was observed that all the variables considered: container, validation, design, incentive, and form were moderators of the value of α. The results are shown in Table 4.

No adjustments were made to the *p*-value that was obtained by the ANOVA tests, due to the very low *p*-values that were obtained (the exact *p*-values obtained were actually *p* < 0.000001). Using any adjustment to the value would not have produced any differences in the results.

### 3.4. Robust Estimate

The robust estimation of the results of the PTM instrument, once the identified outliers were eliminated, can be found in Table 5. In the public subscale, the number of identified outliers was 15, with a mean of *α* of 0.78 (95% CI: 0.76–0.79), reducing its heterogeneity calculated using the *I^2^* index to 56.96, considered medium.

Two other subscales, dire and compliant, also reduced their levels of heterogeneity from high to medium. In the dire subscale, 10 outlier studies were counted which, once eliminated, produced a mean of α for this subscale of 0.73 (95% CI: 0.71–0.75) and a heterogeneity value of 61.59%. The outliers identified in the compliant subscale were 14, once eliminated, the mean of *α* for this subscale was 0.76 (95% CI: 0.74–0.78) and its level of heterogeneity was 53.61%. Finally, the most considerable reduction in the level of heterogeneity occurred in the anonymous subscale, in which 14 outliers were eliminated. Thus, an α mean of 0.81 (95% CI: 0.8–0.81) and an *I^2^* index of 6.31, considered close to null, were obtained.

## 4. Discussion

The objective of this study was to evaluate the characteristics of the reliability reports, the induction, and the reliability generalization of the PTM, whose measurement is widely used for prosocial tendencies, especially in English-speaking users, measuring different prosocial tendencies [19].

Regarding the metric quality of the instruments, reliability constitutes one of the most important psychometric properties when psychological tests are applied to a sample of participants, providing information about the degree of precision of the measurement associated with a test [42], having to report original estimates of the reliability of the tests with the sample data itself [43]. The results of this study showed that only 47.9% of the selected studies reported the reliability index, 38% induced reliability, 7.5% reported imprecisely this property, and 20.95% did not report it at all. Vacha-Haase et al. [39] set out to review the reliability report of three journals (Professional Psychology, Journal of Counseling Psychology, and Psychology & Aging) between 1990 and 1997. From the total of 839 articles, they concluded that only 35.6% provided their reliability coefficients for the study data, 22% induced it from previous studies, and 3.8% referred to the reliability of the instrument in previous studies, with imprecise values. Finally, 36.4% did not even mention the concept of reliability. The comparison of these data with the PTM data shows that in these 20 years there has been an improvement, in general, in the practice of reporting the reliability of an instrument. Historically, 36.4% did not report this data; nowadays, it is only 20.95%. However, it seems insufficient given the importance of this property. On the other hand, around 20% of the studies, both in the study by [42] and in the present study, reported induced reliability. This malpractice has the aggravating circumstance that it can lead the researcher to the false sensation of reporting reliability when, actually, he is not indicating the reliability of the scale in his study. All of it should imply an appeal to the research community to encourage them to proceed with greater rigor, regarding the reporting of this statistical data.

Reliability generalization is a meta-analysis method used to explore the variability in the estimates of this property of the test and characterize the sources of this variance [44]. This must take into account that consistency refers to the scores and not the test, therefore, for each application of the test, one or more reliability coefficients may be established, which may vary due to various factors. For this reason, studying how the reliability coefficients vary in each group, whether normative or not, constitutes a scientific task that the researcher must evaluate [32]. About those indicated, a meta-analysis of generalization was performed regarding the reliability and the calculation of the heterogeneity for the scores of the four mentioned subscales, evaluating the bias in the selection of the articles. The average values of Cronbach’s Alpha reliability were located between 0.74 to 0.80, being the lowest value for the Dire scale and the highest for Anonymous. These magnitudes of the α coefficient are considered a limit based on what was established by [45], and reaffirmed by [46] for exploratory research; they are not adequate for basic research and even less for making important decisions [47].

Regarding the sources of variability of the reliability coefficients, the influence of both a series of continuous variables and categorical variables was analyzed.

First, the results obtained with the analysis of continuous variables showed that none of the subscales had a significant influence on the Public subscale. According to the Anonymous scale, the only significant ones were the average age of the sample and the typical deviation in this value. These results would find justification within psychometric theory, since the greater the heterogeneity in the sample, the greater the reliability coefficient of the applied instrument.

Finally, while only the percentage of women had a significant effect on the heterogeneity of the reliability values in the Dire scale, in the Compliant scale, both the mean age of the sample and the scale mean score would have a significant effect. 

Second, analyzing the categorical moderating variables, it was found that all the variables analyzed (container, validation, design, incentive, and form) had significant effects on the heterogeneity of the reliability coefficients.

The REGEMA guidelines [41] provide a checklist for the authors to corroborate that they are following the necessary steps when performing a meta-analytic report. This checklist appears in Appendix A. This study ensured a good reproducibility, which means that any other researcher could repeat it, following the same steps and calculations, even with the same data [48]. 

### 4.1. Limitations and Future Research

Regarding the limitations, heterogeneity due to the multiple languages, countries that made their own adaptations and different number of items in which PTM was presented should be taken into account. Besides that, it has an original version and a revised version. Not all articles have adequately measured reliability. All of this means that the final sample was composed by a fewer number of articles than those initially obtained in the systematic review. The final number of subjects, the statistical analyses quality, the different languages in which the scale is presented, etc., are also reduced. Furthermore, the studies that are included in this research do not provide enough information about different biases that might be influencing the reliability, which make it difficult to interpret the heterogeneity. Some examples of these biases are the characteristics of the sample or their size, the response patterns of their subjects, etc.

Another limitation of this research is the fact that some levels of the moderator variables have a low representation, which could affect the generalization of the results.

For future research, it would be interesting to expand and review the moderators that can act as variables that make it difficult to generalize the reliability of the instrument. Including a greater number of studies can help with the analysis of the PTM properties and, due to that, the standardization of the results. Repeating this meta-analysis is considered a good indication for the future. 

### 4.2. Practical Implication

Prevention and socio-psychological intervention can be nourished by the study of instruments directed at the study of psychological constructs, as it is the case of prosocial behavior. Increasing prosocial behaviors and decreasing disruptive ones in the general population can be facilitated by the analysis of the PTM and PTM-R instruments. Therefore, psychology professionals can benefit from this study because they obtain a positive assessment of the PTM instrument. With this information, professionals can, for instance, use the instrument to measure the baseline level of prosociality as a pretest before conducting an intervention or as a posttest when the intervention is done or even know the relationship that prosociality can have with other psychological constructs. On the other hand, achieving a proper degree of confidence ensures that the results can be generalized. The random coefficients model is considered an acceptable option for the generalization of the results in futures studies different from this one. Generalization is one of the preferred research objectives [49].

This study does not end in assessing the suitability of the PTM and PTM-R. It is also trying to take its part in and improve the research of reliability standards directed to the instruments used in the healthcare settings. The study of reliability meta-analysis has allowed us to recognize the importance of having equivalent sample groups. Apart from that, there have been previous research focused on making the authors think about which of the reliability coefficients (choosing between *α* and *ω*) was more appropriate for their study [50]. Furthermore, it is encouraged to use an ESEM model instead of the AFC model, or even the use of both, when measuring dimensionality. ESEM is considered more recommended when measuring psychological variables [51,52].

This study also aims to encourage authors to foster a deeper analysis of reliability, reporting its indexes, even when their articles are not uniquely directed to analyze the psychometric properties of an instrument. We believe that it will be helpful for the rest of authors and for the reviewers since the aim is to establish the reliability report in a normative manner. Guidelines [53,54] and organizations [55,56] that promote good practices encourage the researchers to achieve transparency in their works, which is also promoted by this study, especially when it comes to the use of an instrument directed to assess variables in the field of health.

## 5. Conclusions

This research presents new and different ways of analyzing the implementation of the PTM and the PTM-R. The meta-analytic results show that many of the samples of the studies extracted do not provide data that helps with the interpretation of the reliability generalization. Despite this, it is observed that the PTM and PTM-R instruments, in their original version, present good values to be used to measure the prosocial behavior of the general population. It would be interesting, for future research, to know if it would be considered correct to use this instrument for clinical diagnosis, something that is ruled out by the results of this research.

## Figures and Tables

**Table 1:** Main statistics of the 60 studies included in the meta-analysis.

Study	*n*	Sample ^a^	M Age	M	SD	Women%	Language ^b^	Version ^c^	α Total	α PU ^d^	α AN ^d^	α CO ^d^	α DR ^d^
1	182	1	21	NR	NR	76	9	1	0.64	0.71	0.86	0.77	0.69
2	261	1	NR	2.68	2.4	55.2	2	1	0.79	NR	NR	NR	NR
3	897	2	15	3.65	0.60	54.2	2	1	0.94	0.80	0.84	0.79	0.72
4	358	2	15	3.48	0.87	70	1	1	0.90	NR	NR	NR	NR
5	486	3	39	3.17	0.51	57.4	1	1	0.84	0.83	0.88	0.86	0.80
6	110	1	21	NR	NR	50	1	1	0.86	NR	NR	NR	NR
7	416	3	27	NR	NR	37.5	1	1	0.85	NR	NR	NR	NR
8	347	2	NR	NR	NR	46.9	1	1	0.92	NR	NR	NR	NR
9	314	3	32	4.87	0.53	56	2	1	0.79	NR	NR	NR	NR
10	1907	1	20	3.35	0.51	67.3	2	1	0.90	NR	NR	NR	NR
11	269	1	23	3.37	0.41	67.6	2	1	0.82	0.64	0.79	0.82	0.64
12	627	2	10	3.78	0.73	NR	1	1	0.85	NR	NR	NR	NR
13	305	3	27	3.60	0.60	42.6	2	1	0.93	0.84	0.85	0.83	0.76
14	149	3	NR	2.82	0.67	NR	1	1	0.86	NR	NR	NR	NR
14	122	2	NR	2.90	0.70	27	1	1	0.87	NR	NR	NR	NR
15	888	1	21	NR	NR	8.4	1	1	0.81	NR	NR	NR	NR
16	395	1	23	3.37	0.34	63.29	2	1	0.63	NR	NR	NR	NR
17	358	2	13	3.79	0.71	49.72	2	1	0.95	0.85	0.84	0.86	0.85
18	142	2	16	NR	NR	63	1	1	NR	0.85	0.82	0.81	0.76
19	324	1	19	NR	NR	79.6	1	1	NR	0.80	0.82	0.75	0.70
20	203	2	13	NR	NR	5.7	1	1	NR	NR	NR	0.75	NR
21	148	1	23	NR	NR	66.9	1	1	NR	0.86	0.89	0.74	0.76
22	749	2	10	NR	NR	49	1	1	NR	0.75	0.76	0.64	0.76
23	539	1	19	NR	NR	75.5	1	1	NR	0.86	0.83	NR	NR
24	187	1	19	NR	NR	100	1	1	NR	0.74	0.83	0.79	0.66
25	749	2	15	NR	NR	48.1	1	1	NR	NR	NR	0.67	0.78
26	46	1	19	NR	NR	50	1	1	NR	0.85	0.77	0.72	0.84
27	334	2	12	NR	NR	47	1	1	NR	0.78	0.81	0.72	0.78
27	1792	1	NR	NR	NR	NR	1	1	NR	0.80	0.81	0.81	0.73
28	581	3	34	NR	NR	78.3	4	1	NR	0.78	0.81	0.78	0.54
29	1527	1	20	NR	NR	75.2	1	1	NR	0.81	0.80	0.77	0.64
30	126	2	13	NR	NR	4.47	1	1	NR	0.70	0.73	NR	0.70
31	545	3	34	NR	NR	77.6	4	1	NR	0.72	0.81	0.78	0.54
32	148	1	23	NR	NR	67	1	1	NR	NR	NR	NR	NR
33	202	1	20	NR	NR	76.5	1	1	NR	0.75	NR	NR	NR
34	148	2	15	NR	NR	66.89	1	1	NR	NR	NR	0.77	NR
35	398	1	20	NR	NR	73.4	1	1	NR	0.83	NR	NR	NR
36	186	1	21	NR	NR	78.5	1	1	NR	NR	NR	0.78	0.83
37	412	1	21	NR	NR	5.97	5	1	NR	NR	0.72	0.69	NR
38	140	1	19	NR	NR	4.71	5	1	NR	0.62	0.80	0.68	NR
38	117	1	18	NR	NR	64.10	1	1	NR	0.77	0.70	0.85	NR
39	1018	1	21	NR	NR	83.49	7	1	NR	NR	NR	NR	NR
40	438	1, 2	NR	NR	NR	NR	5	1	NR	NR	0.81	NR	NR
41	435	3	34	NR	NR	61.4	6	1	NR	0.70	NR	NR	NR
42	80	2	14	NR	NR	61.25	1	2	NR	0.76	0.76	0.80	0.71
42	58	2	17	NR	NR	53.44	1	2	NR	0.86	0.84	0.75	0.75
43	207	2	10	NR	NR	50	1	2	NR	0.74	0.69	0.51	0.69
44	207	2	10	NR	NR	51	1	2	NR	0.74	0.69	0.51	0.69
44	108	2	11	NR	NR	50	1	2	NR	0.77	0.71	0.62	0.62
45	57	3	23	NR	NR	50	8	2	NR	NR	0.67	NR	NR
46	233	2	16	NR	NR	69	1	2	NR	0.83	0.86	0.67	0.82
47	311	2	16	NR	NR	58.7	1	2	NR	0.64	0.74	0.77	0.76
48	140	2	16	NR	NR	64	1	2	NR	0.77	0.77	0.65	0.82
49	904	2	12	NR	NR	48.67	1	2	NR	0.70	0.76	0.63	0.82
50	207	2	10	NR	NR	51	1	2	NR	0.78	0.75	0.51	0.69
51	302	2	14	NR	NR	46.7	1	2	NR	0.84	NR	NR	NR
52	302	2	14	NR	NR	46.7	1	2	NR	0.84	0.80	0.53	0.77
53	265	2	14	NR	NR	62	3	2	NR	0.77	0.74	0.42	0.65
54	35	2	16	3.31	0.58	47.5	3	2	0.79	NR	NR	NR	NR
55	240	2	14	NR	NR	57.9	2	2	NR	0.73	0.80	0.82	0.68
56	187	1	18	NR	NR	49	1	2	NR	0.80	0.79	0.84	0.69
57	311	2	16	NR	NR	58.7	1	2	NR	0.64	NR	0.77	0.76
58	202	1	20	NR	NR	76.5	1	2	NR	0.75	0.80	0.71	0.76
59	253	1	21	3.69	0.86	58.2	1	2	NR	NR	NR	0.80	0.82
60	189	1	18	NR	NR	49	1	2	NR	0.80	0.79	0.84	0.69

^a^ Type of sample: 1 = University students; 2 = Adolescents; 3 = Adults. ^b^ Scale language: 1 = English; 2 = Chinese; 3 = Spanish; 4 = Serbian; 5 = Turkish; 6 = Italian; 7 = Greek; 8 = German; 9 = Persian. ^c^ Version of the scale: 1 = PTM; 2 = PTM-R. ^d^ Subscales: PU = Public; AN = Anonymous; CO = Compliant; DR = Dire.

**Table 2:** Estimates for PTM reliability scores.

Scores (*k*)	*α* Means		Heterogeneity			
	*α*	CI 95%	*τ*^2^ (*τ*)	*Q* (df)	I%	H
**Public (41)**	0.78	0.76, 0.80	0.065	341.56 ** (40)	89.51	9.53
**Anonymous (39)**	0.80	0.79, 0.82	0.048	240.11 ** (38)	87.11	7.76
**Dire (38)**	0.74	0.71, 0.76	0.070	366.79 ** (37)	89.33	9.37
**Compliant (41)**	0.75	0.72, 0.78	0.115	395.60 ** (40)	90.55	10.58

*k*: number of items. *Q*: statistical test for heterogeneity. I%: percentage of heterogeneity. H: excess of *Q* value in case heterogeneity did not exist. τ2 (τ): variability estimator; ** *p* < 0.01.

**Table 3:** Analysis of continuous moderating variables.

IV (*k*)	*b*	CI(95%)	*Q_M_*	*p*	*Q_E_*	*R* ^2^
Public score						
Year of publication (41)	−0.01	−0.03, 0.01	1.98	0.17	341.40 ***	1.99%
Sample year (5)	−0.01	−0.05, 0.03	0.44	0.56	7.78	0%
Age (mean) (40)	0.01	−0.01, 0.92	0.12	0.73	333.82 ***	0%
Age (SD) (33)	0.001	−0.03, 0.03	0.005	0.95	217.20 ***	0%
Average score (25)	0.06	−0.02, 0.14	2.23	0.15	161.33 ***	7.21%
SD score (25)	0.17	−0.02, 0.37	3.30	0.08	151.91 ***	12.04%
Percentage of women (40)	0.001	−0.01, 0.01	0.06	0.80	327.80 ***	0%
IPS Ranking (41)	0.001	−0.004, 0.002	0.82	0.37	340.21 ***	0%
Anonymous score						
Age (mean) (37)	0.02 **	0.01, 0.03	11.45	0.002	182.65 ***	27.1%
Age (SD) (32)	0.03 *	0.003, 0.06	4.95	0.03	169.61 ***	13.79%
Percentage women (36)	0.01	−0.001, 0.01	3.35	0.08	224,005 ***	6.34%
SD score (22)	0.08	−0.07, 0.23	1.24	0.28	94.13 ***	2.4%
Year of publication (39)	−0.004	−0.02, 0.01	0.30	0.59	237.43 ***	0%
Sample year (5)	0.01	−0.03, 0.04	0.35	0.60	6.47	0%
Average score (22)	0.01	−0.03, 0.05	0.43	0.52	99.31 ***	0%
IPS Ranking (33)	−0.001	−0.005, 0.003	0.36	0.55	196.96 ***	0%
Dire score						
Year of publication (38)	0.01	−0.02, 0.01	0.21	0.65	331.94 ***	0%
Sample year (6)	0.01	−0.06, 0.01	3.04	0.16	28.16 ***	29.19%
Age (mean) (37)	0.01	−0.02, 0.004	2.03	0.16	308.28 ***	4.33%
Age (SD) (30)	−0.03	−0.06, 0.01	2.14	0.15	245.16 ***	4.04%
Average score (24)	0.15	−0.27, 0.36	0.08	0.78	205.28 ***	0%
SD score (24)	0.04	−0.91, 0.99	0.01	0.93	203.93 ***	0%
Percentage of women (37)	0.004 *	−0.01, 0.001	4.69	0.04	239.38 ***	12.87%
IPS Ranking (38)	0.002	−0.01, 0.002	1.21	0.28	359.58 ***	0.64%
Compliant score						
Year of publication (41)	0.01	−0.02, 0.03	0.40	0.53	352.82 ***	0%
Sample year (6)	0.01	−0.07, 0.09	0.11	0.76	45.59 ***	0%
Age (mean) (40)	0.01 ***	0.01, 0.04	12.73	0.001	242.34 ***	26.99%
Age (SD) (33)	0.04	−0.01, 0.08	3.12	0.09	215.36 ***	6.83%
Average score (26)	0.44 *	0.11, 0.77	7.36	0.01	125.74 ***	26.64%
SD score (26)	−0.66	−1.74, 0.42	1.61	0.22	181.82 ***	3.62%
Percentage of women (40)	0.01	−0.003, 0.02	1.82	0.19	328.58 ***	2%
IPS Ranking (41)	0.002	−0.003, 0.01	0.80	0.38	394.25 ***	0%

* *p* < 0.01; ** *p* < 0.001; *** *p* < 0.0001. b = regression coefficient of the moderating variable. *Q_M_* = statistical to test the statistical significance of the moderating variable. *QE* = statistical to check if the model is well specified. *R*2 = proportion of the variance explained by the moderating variable.

**Table 4:** Analysis of categorical moderating variables.

IV (*k*)		*α*	CI 95%	*p*	*Q_W_*	*Q.B.*
Public score						
Validation (40)	Original (30)	0.79	0.77, 0.81	<0.001	300.10 ***	404.66 ***
	Free translation (4)	0.73	0.62, 0.81	<0.001		
	Validated version (6)	0.77	0.67, 0.85	<0.001		
Continent (41)	Asia (7)	0.77	0.70, 0.83	<0.001	309.27 ***	312.77 ***
	Central America (1)	0.77	0.72, 0.81	<0.001		
	Europe (4)	0.72	0.60, 0.80	<0.001		
	North America (29)	0.79	0.77, 0.81	<0.001		
Design (38)	Longitudinal (3)	0.78	0.57, 0.89	<0.001	328.91 ***	541.17 ***
	Cross (35)	0.78	0.75, 0.80	<0.001		
Incentive (39)	Credits (10)	0.81	0.77, 0.84	<0.001	246.81 ***	248.39 ***
	Economic (8)	0.80	0.76, 0.84	<0.001		
	No incentive (18)	0.75	0.71, 0.78	<0.001		
	Gift (2)	0.74	0.58, 0.84	<0.001		
	Unspecified Reward (1)	0.75	0.72, 0.78	<0.001		
Shape (37)	Online (9)	0.80	0.68, 0.87	<0.001	318.08 ***	496.21 ***
	Paper (28)	0.89	0.83, 0.92	<0.001		
Anonymous score						
Continent (39)	Asia (7)	0.82	0.77, 0.86	<0.001	211.44 ***	413.86 ***
	Central America (1)	0.74	0.69, 0.79	<0.001		
	Europe (5)	0.79	0.74, 0.83	<0.001		
	North America (26)	0.80	0.78, 0.82	<0.001		
Validation (39)	Original (27)	0.80	0.78, 0.82	<0.001	229.32 ***	550.15 ***
	Free translation (4)	0.79	0.74, 0.83	<0.001		
	Validated version (8)	0.82	0.78, 0.85	<0.001		
Design (39)	Longitudinal (2)	0.81	0.48, 0.93	<0.001	239.79 ***	818.03 ***
	Cross (37)	0.80	0.78, 0.82	<0.001		
Incentive (36)	Credits (8)	0.83	0.79, 0.86	<0.001	198.85 ***	310.27 ***
	Economy (7)	0.79	0.71, 0.84	<0.001		
	No incentive (18)	0.80	0.78, 0.82	<0.001		
	Gift (2)	0.80	0.80, 0.80	<0.001		
	Unspecified Reward (1)	0.76	0.73, 0.79	<0.001		
Shape (34)	Online (8)	0.81	0.78, 0.84	<0.001	212.30 ***	711.34 ***
Dire score						
Continent (38)	Asia (6)	0.73	0.63, 0.81	<0.001	240.08 ***	265.08 ***
	Central America (1)	0.65	0.57, 0.72	<0.001		
	Europe (2)	0.54	0.54−0.54	<0.001		
	North America (29)	0.75	0.73, 0.77	<0.001		
Validation (38)	Original (29)	0.75	0.73, 0.77	<0.001	237.88 ***	372.72 ***
	Free translation (4)	0.59	0.48, 0.68	<0.001		
	Validated version (5)	0.75	0.63, 0.83	<0.001		
Design (35)	Longitudinal (3)	0.75	0.55, 0.86	<0.001	345.34 ***	351.30 ***
	Cross (32)	0.73	0.70, 0.76	<0.001		
Incentive (36)	Credits (9)	0.73	0.66, 0.79	<0.001	330.59 ***	132.88 ***
	Economic (8)	0.75	0.69, 0.80	<0.001		
	No incentive (15)	0.72	0.66, 0.77	<0.001		
	Gift (3)	0.76	0.51, 0.88	<0.001		
	Unspecified Reward (1)	0.76	0.73, 0.79	<0.001		
Shape (34)	Online (8)	0.73	0.62, 0.81	<0.001	304.04 ***	348.13 ***
	Paper (26)	0.74	0.72, 0.77	<0.001		
Compliant score						
Continent (41)	Asia (6)	0.83	0.80, 0.86	<0.001	294.05 ***	201.21 ***
	Central America (1)	0.42	0.26, 0.54	0.09		
	Europe (4)	0.74	0.64, 0.81	<0.001		
	North America (30)	0.74	0.71, 0.77	<0.001		
Validation (41)	Original (31)	0.75	0.71, 0.78	<0.001	382.15 ***	195.05 ***
	Free translation (4)	0.73	0.39, 0.88	<0.001		
	Validated version (6)	0.79	0.70, 0.85	<0.001		
Design (38)	Longitudinal (3)	0.68	0.14, 0.88	<0.001	330.94 ***	308.81 ***
	Cross (35)	0.76	0.73, 0.79	<0.001		
Incentive (38)	Credits (9)	0.78	0.73, 0.82	<0.001	302.25 ***	113.46 ***
	Economic (8)	0.68	0.53, 0.79	<0.001		
	No incentive (17)	0.76	0.71, 0.80	<0.001		
	Gift (3)	0.78	0.60, 0.88	<0.001		
	Unspecified incentive (1)	0.64	0.58, 0.69	0.005		
Shape (36)	Online (8)	0.75	0.66, 0.82	<0.001	335.94 ***	243.91 ***
	Paper (28)	0.75	0.71, 0.78	<0.001		

*** *p* < 0.0001. *b* = regression coefficient of the moderating variable. *Q_W_* = statistical to test the statistical significance of the moderating variable. *Q.B.* = statistical to check if the model is well specified. *R*^2^ = proportion of the variance explained by the moderating variable.

**Table 5:** Robust estimates (without outliers) for PTM scores.

Scores	*n* Outliers (%)	*α* Means		Heterogeneity				
		*α* (se)	CI 95%	% Atten.	*τ*^2^ (*τ*)	*Q* (df)	I%	H
Public	15	0.78 ** (0.03)	(0.76, 0.79)	−22.17	0.010 (0.10)	58.62 ** (25)	56.96	2.32
Anonymous	14	0.81 ** (0.02)	(0.80, 0.81)	−20.29	0.0005 (0.02)	32.97 ** (24)	6.31	1.07
Dire	10	0.73 ** (0.03)	(0.71, 0.75)	−25.78	0.016 (0.12)	65.56 ** (27)	61.59	2.60
Compliant	14	0.76 ** (0.04)	(0.74, 0.78)	−25.91	0.017 (0.13)	53.40 ** (26)	53.61	2.16

Note. % atten: attenuation percentage: 100(alphawith outliers − alphawithout outliers/alphawith outliers). Studies identified as outliers (see Table 1 for numbering): Public = “4”, “5”, “6”, “7”, “8”, “10”, “12”, “20”, “22”, “23”, “25”, “31”, “33”, “35”, “39”. Anonymous = “2”, “3”, “4”, “6”, “10”, “11”, “21”, “23”, “27”, “28”, “29”, “30”, “32”, “35”. Dire = “4”, “5”, “7”, “17”, “18”, “20”, “21”, “26”, “29”, “37”. Compliant = “3”, “5”, “6”, “11”, “13”, “16”, “24”, “27”, “32”, “33”, “34”, “35”, “37”, “41”; * *p* < 0.05; ** *p* < 0.01.

## Data Availability

Analysis script is available on request from the authors.

## Appendix A

**Table A1:** Checklist for the corroboration of the meta-analytic report according to the method.

TITLE		Yes	No	Page	NA
1. Title	In the title include: (a) the term “reliability generalization” or “meta-analysis” together with some explicit indication to reliability (internal consistency, test–retest, inter- or intra-rater) and (b) the name of the scale or, if more than one scale, the attribute/outcome measure that the scales are assessing.	X		1	
ABSTRACT		Yes	No	Page	NA
2. Abstract	In the abstract explicitly state: (a) that the objective was to carry out a reliability generalization (RG) meta-analysis of one or several scales; (b) eligibility criteria of the studies; (c) data sources with the temporal range covered; (d) types of reliability coefficients analyzed; (e) statistical model applied; (f) main results (e.g., pooled reliability coefficient and 95% CI, moderator variables related to reliability); and (g) main conclusions. In case of space limitation, (b) and (c) criteria can be omitted.	X		1	
INTRODUCTION		Yes	No	Page	NA
3. Background	In the background include: (a) a conceptual definition of the attribute/outcome measure assessed by the scale/s; (b) description of the target population/s to which the scale/s is/are applied and its/their purposes (e.g., screening, clinical diagnosis); (c) a complete description of the scale/s (length, number of categories), including the versions and adaptations to other languages/cultures; and (d) a brief presentation of reliability estimates obtained in previous psychometric studies of the scale/s. Optionally, a brief review of validation studies of the scale/s (e.g., exploratory/confirmatory factor analyses, concurrent/convergent/discriminant validity, responsiveness) could be included.	X		2	
4. Objectives	State whether the purpose of the meta-analysis was to obtain a more precise overall reliability coefficient estimate and/or investigate how reliability coefficients vary among different applications of the scales. Optionally, specify whether one objective of the meta-analysis is to estimate the reliability induction rates of the scale/s.	X		5	
METHOD		Yes	No	Page	NA
5. Selection criteria	Specify inclusion criteria: (a) name/s of the scale/s analysed in the RG meta-analysis, as well as the versions and/or adaptations included; (b) geographical and/or cultural restrictions; (c) years considered; (d) language of the paper; (e) publication status; (f) to report any reliability estimate based on the study-specific sample/s; (g) type/s of reliability considered (e.g., internal consistency, temporal stability, inter-/intra rater reliability…); (h) target population/s (e.g., community, clinical, subclinical/analog, university…); and (i) minimum sample size required.	X		7	
6. Search strategies	Specify how the studies were located: (a) electronic databases consulted; (b) other formal search procedures (e.g., manual search in specific journals, backward search from references listed in selected studies); and (c) informal search procedures (e.g., internet searches, contacting study authors to identify additional studies). For electronic searches, describe the search strategy, including the keywords used and how they were combined, and the search limits (e.g., fields where the keywords were searched—title, abstract, full-text, temporal range, language).	X		5	
7. Data extraction	Describe the characteristics extracted from the studies, including: (a) sample size/s, mean/s and standard deviation/s of total test scores and subscales (if applicable); (b) sample characteristics (e.g., target population, country, mean age, standard deviation of the age, gender distribution, ethnic distribution, disorder history—mean and SD in years); (c) test version (e.g., adaptation/version, number of items, reporting format—self-report, clinician); (d) methods (e.g., study design, purpose of the study—psychometric versus applied, quality checklist); (e) extrinsic characteristics (e.g., publication status, researchers’ affiliations, funding source).	X		7	
8. Reported reliability	Identify the types of reliability coefficients included in the RG meta-analysis: internal consistency (e.g., Cronbach’s alpha, KR-21, parallel forms, omega), temporal stability (test–retest), inter- and intra-rater reliability (e.g., intraclass correlation, kappa coefficient). Clearly state that separate meta-analyses were conducted for each type of reliability coefficient. In case of applying a multivariate/MASEM approach, specify the type of statistical information extracted from the studies (i.e., item–item correlation/covariance matrices, factor loadings, etc.).	X		7	
9. Estimating the reliability induction and other sources of bias	In case that the meta-analysis intends to estimate the reliability induction, identify the types of reliability induction: induction by omission (no mention of test reliability whatsoever) or reporting induction (vague or precise reporting). Describe how other sources of bias were assessed (e.g., assumptions of the reliability coefficient, adequacy of the measurement model, etc.).	X		7	
10. Data extraction of inducing studies	Declare whether characteristics of inducing studies were also extracted or if, on the contrary, only characteristics of studies that reported reliability were extracted.	X		7	
11. Reliability of data extraction	Describe how the reliability of data extraction process was appraised: how many coders which agreement coefficients were applied (e.g., kappa coefficient, intraclass correlation), which values were obtained, and how disagreements were dealt with.	X		8	
12. Transformation method	State whether or not the reliability coefficients were transformed for the meta-analytic integration. If relevant, specify the transformation methods: Fisher’s Z for correlation coefficients (e.g., test–retest coefficients), Bonett’s and Hakstian and Whallen’s transformation for internal consistency coefficients (e.g., Cronbach’s alpha), reliability index, measurement error (e.g., standard error of measurement), or other (specify).	X		9	
13. Statistical model	Describe the statistical model(s) assumed in the meta-analytic integration for estimating the average reliability coefficient and for analysing the influence of moderator variables (e.g., fixed-effect(s), random-effects, mixed-effects, varying-coefficient models, generalized linear models), as well as the analysis framework (frequentist or Bayesian). In case of applying a multivariate/MASEM approach, describe how the item correlation/covariance matrices or factor loadings were synthesized.	X		10	
14. Weighting method	Specify the weighting method applied in the meta-analytic integration: unweighted, weighting by sample size, weighting by inverse variance, or other weighting methods.	X		10	
15. Heterogeneity assessment	Describe how heterogeneity among reliability coefficients was assessed (e.g., standard deviation, *Q* statistic, *I^2^* index, between-studies variance, 75% rule of Hunter-Schmidt). If relevant, specify the between-studies variance estimator (DerSimonian and Laird, Maximum Likelihood, Restricted Maximum Likelihood, Empirical Bayes, Paule and Mandel), as well as how confidence intervals, credibility intervals, or prediction intervals were calculated.	X		10	
16. Moderator analyses	If relevant, describe how the influence of moderator variables was assessed (e.g., subgroup analyses, meta-regression analyses, correlational analyses).	X		10	
17. Additional analyses	Describe other additional analyses accomplished, such as sensitivity analyses (e.g., statistical analyses with transformed and untransformed reliability coefficients, one-to-one deleting of reliability coefficients, assessment of publication bias, reporting biases, and other sources of bias).	X		10	
18. Software	Mention the software and version used to carry out the statistical analyses (e.g., metafor in R, Proc MIXED in SAS, Comprehensive Meta-analysis).	X		10	
RESULTS		Yes	No	Page	NA
19. Results of the study selection process	Describe, ideally with a flow chart, the selection process of the studies, specifying the number of studies identified from each search source, excluded studies and reasons why, and the number of studies that reported and induced reliability of test scores. Regarding reliability induction, report induction rates, distinguishing between induction “by omission” and “by report” (see e.g., REGEMA flowchart). Furthermore, it is advisable to compare the reliability induction rates as a function of variables such as publication year, country/continent and study purpose (psychometric vs. applied).	X		6	
20. Mean reliability and heterogeneity	Present pooled reliability coefficients and confidence/credibility intervals for the scale (and subscales, if applicable) and for each type of reliability (e.g., internal consistency, temporal stability, inter- and intra-rater agreement). In case of applying any transformation of the reliability coefficients, results should be back-transformed to the original metric to facilitate interpretation. Illustrate the distribution of reliability coefficients with graphical techniques (e.g., forest plots, box plots, stem and leaf displays, histograms) and describe the degree of heterogeneity by one or more heterogeneity measures (see Item 15).	X		10	
21. Moderator analyses	For categorical moderators, provide the pooled reliability coefficient, confidence interval and other heterogeneity measures for each category of the moderator. For continuous moderators, include the regression coefficients, standard errors and confidence limits. For both types of moderators, report results of the statistical significance tests, misspecification tests, and proportion of variance accounted for. As a further step, it is advisable to fit a predictive/explanatory model including the most relevant moderator variables.	X		10	
22. Sensitivity analyses	Report or describe the results of any sensitivity analyses conducted (see Item 17).	X		13	
23. Comparison of inducing and reporting studies	If performed, present the results of comparing the characteristics of inducing and reporting studies (e.g., sociodemographic and clinical characteristics of the samples).	X		8	
24. Data set	Tabulate the characteristics of the individual studies that reported reliability (see Item 7). Tables can be presented as appendices or supplementary files. In addition, list of all studies included in the RG meta-analysis, either in the reference section or as a supplementary file.	X		8	
DISCUSSION		Yes	No	Page	NA
25. Summary of results	Present the main results, such as mean reliability exhibited by the scale/test and moderators of the reliability coefficients. If available, discuss the results in the light of previous evidence.	X		14	
26. Limitations	Discuss the limitations of the meta-analysis. Include an explicit statement of the reliability induction rates and the extent to which inducing and reporting studies are comparable in terms of samples characteristics.	X		15	
27. Implications for practice	Provide guidelines for professional practice regarding the usefulness of the scale/test in different settings and target populations.	X		15	
28. Implications for future research	Include recommendations for researchers regarding the conditions under which the scale/test should be applied.	X		15	
FUNDING		Yes	No	Page	NA
29. Funding	State the financial sources of the meta-analysis, as well as potential conflict of interests of the authors.	X		17	
PROTOCOL		Yes	No	Page	NA
30. Protocol	State whether a protocol of the meta-analysis was previously published or made accessible in some web-site (e.g., in Prospero).				X

NA: Not Applicable.

## Appendix B

**Table A2:** Numbering of the studies included in the meta-analysis.

Study	Author, Year
1 [26]	Azimpour, 2012
2 [57]	Meng & Meng, 2020
3 [58]	Yu et al., 2020
4 [59]	Rious et al., 2019
5 [60]	Ng et al.,2018
6 [61]	Lockwood et al., 2014
7 [62]	Collins & Freeman, 2013
8 [63]	Schwartz et al., 2007
9 [64]	Bao et al.,2020
10 [65]	Lin et al., 2021
11 [66]	Guan et al., 2019
12 [67]	White et al., 2018
13 [68]	Huang et al., 2016
14 [69]	Kauten & Barry, 2015
15 [70]	Schwar & Mahony, 2012
16 [71]	Shi et al., 2020
17 [72]	Yu et al., 2018
18 [21]	Hardy & Carlo, 2005
19 [73]	Mc Ginley et al., 2021
20 [74]	Laible et al., 2010
21 [75]	Carlo et al., 2012
22 [76]	Brittian et al., 2013
23 [77]	White, 2014
24 [78]	Davis et al., 2016
25 [79]	Carlo et al., 2018
26 [80]	Morelli et al., 2018
27 [81]	Vaughan et al., 2020
28 [82]	Dinic & Bodroza, 2021
29 [83]	Davis et al., 2017
30 [84]	De Guzmán et al., 2012
31 [82]	Dinic & Bodroza, 2020
32 [85]	Memmott et al., 2020
33 [86]	Davis, 2020
34 [87]	Laible et al., 2014
35 [88]	Christ et al., 2016
36 [89]	Streit et al., 2018
37 [90]	Kindap & Aktas, 2019
38 [91]	Gülseven et al., 2020
38 [92]	Gülseven et al., 2021
39 [93]	Kornilaki, 2021
40 [94]	Bayraktar et al., 2009
41 [30]	Castiglioni et al., 2019
42 [20]	Carlo et al., 2003
43 [95]	Davis et al., 2015
44 [96]	Carlo et al., 2011
45 [97]	Rodrigues et al., 2018
46 [98]	Carlo et al., 2007
47 [99]	Davis & Carlo, 2019
48 [100]	Hardy et al., 2008
49 [101]	Carlo et al., 2010
50 [102]	Armenta et al.,2011
51 [103]	Davis et al., 2016
52 [104]	McGinley et al., 2021
53 [92]	Gülseven & Carlo, 2021
54 [105]	Gómez-Tabares, 2019
55 [106]	Fu et al., 2015
56 [107]	McGinley, 2018
57 [108]	Davis & Carlo, 2018
58 [109]	Davis et al., 2019
59 [110]	Streit et al., 2020
60 [111]	McGinley, 2020
